# Fatty acids increase neuronal hypertrophy of Pten knockdown neurons

**DOI:** 10.3389/fnmol.2014.00030

**Published:** 2014-04-23

**Authors:** Catherine J. Fricano, Tyrone DeSpenza, Paul W. Frazel, Meijie Li, A. James O'Malley, Gary L. Westbrook, Bryan W. Luikart

**Affiliations:** ^1^Department of Physiology and Neurobiology, Geisel School of Medicine at DartmouthLebanon, NH, USA; ^2^The Dartmouth Institute for Health Policy and Clinical Practice, Geisel School of Medicine at DartmouthLebanon, NH, USA; ^3^The Vollum Institute, Oregon Health and Science UniversityPortland, OR, USA

**Keywords:** Pten, mTOR, pS6, fatty acids, neuronal hypertrophy, PI3K, rapamycin

## Abstract

Phosphatase and tensin homolog (Pten) catalyzes the reverse reaction of PI3K by dephosphorylating PIP3 to PIP2. This negatively regulates downstream Akt/mTOR/S6 signaling resulting in decreased cellular growth and proliferation. Co-injection of a lentivirus knocking Pten down with a control lentivirus allows us to compare the effects of Pten knockdown between individual neurons within the same animal. We find that knockdown of Pten results in neuronal hypertrophy by 21 days post-injection. This neuronal hypertrophy is correlated with increased p-S6 and p-mTOR in individual neurons. We used this system to test whether an environmental factor that has been implicated in cellular hypertrophy could influence the severity of the Pten knockdown-induced hypertrophy. Implantation of mini-osmotic pumps delivering fatty acids results in increased neuronal hypertrophy and p-S6/p-mTOR staining. These hypertrophic effects were reversed in response to rapamycin treatment. However, we did not observe a similar increase in hypertrophy in response to dietary manipulations of fatty acids. Thus, we conclude that by driving growth signaling with fatty acids and knocking down a critical regulator of growth, Pten, we are able to observe an additive morphological phenotype of increased soma size mediated by the mTOR pathway.

## Introduction

Phosphatase and tensin homolog on chromosome 10 (Pten), a phosphatase catalyzing the reverse reaction of phosphatidylinositol 3-kinases (PI3K), cleaves phosphate from phosphatidylinositol (3,4,5)-triphosphate to yield phosphatidylinositol (4,5)-bisphosphate. Thus, Pten inhibits signaling through the Akt/mTOR/S6 pathway downstream of growth factor receptors (Laplante and Sabatini, 2012). Pten dysfunction in mouse neurons results in neuronal hypertrophy and increased excitatory synaptic connectivity (Kwon et al., 2001, 2006; Luikart et al., 2011b; Xiong et al., 2012). The neuronal hypertrophy seen in mouse models parallels the macrocephaly that characterizes a subset of patients with *Pten* mutations and Bannayan-Riley-Ruvalcaba syndrome, Cowden Syndrome, and Autism Spectrum Disorder (ASD) (Longy et al., 1998; Goffin et al., 2001; Butler et al., 2005; McBride et al., 2010).

Further, a number of other proteins related to growth factor signaling including Neurofibromin 1 (NF1), Tuberous Sclerosis Complex 1 and 2 (TSC1/2), and fragile X mental retardation protein (FMRP) are associated with ASD and other co-morbidities such as epilepsy, mental retardation, and tumor formation (Kelleher and Bear, 2008; Bourgeron, 2009). Single gene mutations are estimated to account for 10–20% of ASD cases with no single gene accounting for more than 5% of all ASD cases (Abrahams and Geschwind, 2008; Kelleher and Bear, 2008). Multifactorial genetic and environmental factors likely contribute to the etiology of ASD (Persico and Bourgeron, 2006), however, little is known about how gene/gene and gene/environment interactions influence the expression of cellular phenotypes.

Circulating fatty acids increase p-Akt and p-mTOR signaling in liver and muscle cells (Cao et al., 2008; Riquelme et al., 2011). It is unknown whether a similar signaling change could occur in the brain. Although levels of circulating fatty acids are normally under tight metabolic regulation, increased postprandial levels are observed in individuals with obesity and diabetes (Guo et al., 1999; Labbe et al., 2011). Gestational diabetes and maternal obesity have also been identified as risk factors for ASD (Gardener et al., 2009, 2011; Krakowiak et al., 2012). Thus, we asked whether fatty acids can regulate neuronal hypertrophy and signaling in the context of *Pten* knockdown. Here, we demonstrate that infusion of fatty acids into adult animals increased hypertrophy and pS6/p-mTOR levels in *Pten* knockdown neurons. This hypertrophy was inhibited with the mTOR inhibitor, rapamycin. Thus, direct infusion of fatty acids can influence the expression of cellular phenotypes in the context of a functional *Pten* genetic deficit. It is unknown whether more physiologically relevant situations could produce a similar synergistic effect.

## Materials and methods

All protocols were approved by the Institutional Biosafety and Institutional Animal Care and Use Committee review boards.

### Adult mice viral injections

Six to 8 week old C57BL/6 female mice were anesthetized using an isoflurane gas system (Matrix VIP 3000 isoflourane vaporizer). Females were used in order to decrease the chances of the animals removing their stitches. Viruses were injected into the dentate gyrus using a stereotaxic frame fitted to the anesthesia system. After treating the shaved head with betadine and lidocaine (2.5% lidocaine and 2.5% prilocaine cream), a sagittal incision was made and holes were drilled into the skull at *x*, ±1.1 mm, and *y*, −1.9 mm from bregma. A 10 μ l Hamilton syringe fitted with a 30 gauge needle was used to inject at *z*-depths of −2.5 mm and −2.3 mm. Using the Quintessential Stereotaxic Injector (Stoelting), 1 μ l of virus was delivered to each *z*-depth at a rate of 0.3 μ l/min to a total of 2 μ l of virus per hemisphere. After the injections were complete, the needle was kept in place for 1 min, and then slowly removed. The incision was closed using sutures and treated with betadine, antibiotic ointment, and lidocaine. 80 μ l of ketaprofen (1 mg/mL) were injected intraperitoneally.

### Cloning and viral packaging

All vectors and detailed.gb vector maps are available on request. The viral packaging procedure was as previously described (Luikart et al., 2011b). Viruses were purified by filtering viral supernatant through a 0.45 μm low protein binding syringe tip filter and 5× PEG6000 solution was added to the supernatant to a final concentration of 1× (final concentration is 8% PEG6000 and 0.3M NaCl). This was then mixed gently and incubated at 4°C for 12+h (remixing occasionally). The virus-containing PEG solution was centrifuged at 2500 g for 45 min, the supernatant then removed, and the tube spun again for 2 min to remove all the remaining supernatant. The pellet was resuspended by adding 1/100th the original volume with PBS and placed on a shaker at RT for 30 min.

### Serum fatty acid detection

One hundred to 300 ul of blood was collected into 1.5 mL tubes from adult mice by submandibular bleeding. This blood was left to clot overnight at 4°C, and then spun at 6000 RPM for 20 min at 4°C in order to separate the clot from the serum. 5 ul of each serum sample was used to detect the total levels of free fatty acids using a serum/plasma fatty acid detection kit (Zenbio, Cat # SFA-1). In order to control for any hemolysis that could affect the assay by altering the color of the serum, 5 ul of each sample was diluted up to the total volume required for the assay using the supplied dilution buffer. The optical density of each sample in dilution buffer was subtracted from the optical density obtained from running the assay.

### Filling and implantation of mini-osmotic pumps

Adult mice were injected with mCherry control and GFP shPten lentiviruses in the dentate gyrus. Seven days later, osmotic pumps were implanted subcutaneously. The pumps were prepared as follows: palmitic, myristic, and palmitoleic acids (Sigma) were each dissolved in 100% DMSO at a concentration of 14 mM. Then, 40% ethanol in water was slowly added to each solution 1:1, to make final stock concentrations of 7 mM in 50% DMSO and 20% ethanol. These 7 mM stocks were combined 1:6:16 (palmitoleic: myristic: palmitic) and loaded into the pumps (Alzet, Model 2001). The mini-osmotic pumps, which deliver one microliter of solution per hour for one week, were filled according to the manufacturer's instructions in a sterile environment. The vehicle pumps were filled with 50% DMSO and 20% ethanol in water. Once the pumps were filled, they were primed in 9% sterile saline for 6–12 h at 37°C. Adult mice were anesthetized with the isoflourane system and their backs shaved and prepped with lidocaine and betadine. A small incision was made between the scapulae and the pump was inserted subcutaneously. The incision was then closed with sutures and veterinary glue and 80 μ l of ketaprofen (1 mg/mL) was injected intraperitionally. Finally, the incision was treated with lidocaine, betadine, and antibiotic ointment. 7–10 days later, the mice were perfused and their brains sectioned for analysis. The pumps were removed in order to verify that they had emptied.

### Composition of fatty acid adjusted (FAD) and fatty acid adjusted, high fat (FA-HFD) diets

The FAD consisted of pellets containing 17.7% protein, 60.1% carbohydrate, 7.2% fat by weight plus palmitoleic:myristic:palmitic acids in the ratio 1:6:16 (Modified AIN-93G diet, Harlan Laboratories). The FA-HFD consisted pellets containing 21.0% protein, 44.9% carbohydrate, 20.3% fat plus palmitoleic:myristic:palmitic acids in ratio 1:6:16 (Modified TD.120122 diet, Harlan Laboratories).

### Rapamycin treatment

Adult mice were injected with mCherry control and GFP shPten lentiviruses and implanted 7 days later with miniosmotic pumps containing a mixture of palmitic, palmitoleic, and myristic acids as described above. One day after pump implantation mice were interperitoneally injected daily for 10 days with 10 mg/kg rapamycin. A 25 mg/mL stock solution of rapamycin was prepared in ethanol and a working solution of 1 mg/mL in 4% ethanol, 5% Tween-80 and 5% PEG400 was prepared daily. On the 10th day of injections mice were perfused and brain sections were prepared for confocal analysis.

### Histology

Mice were anesthetized by injecting 600 μ l of 2% Avertin in water intraperitoneally and then perfused with cold PBS with 4% sucrose, then 4% paraformaldehyde(PFA)/ 4% sucrose in PBS through the vasculature of the mouse. After 25 mL of PFA/sucrose were pumped into the mouse, the brains were removed and fixed overnight in PFA/sucrose at 4°C. The next day the brains were washed with PBS sucrose, mounted in 2.5% agarose, and sectioned into 40 μm thick coronal sections using a vibratome (Leica VT 1200 S). For soma size quantitation, the slices containing the hippocampus were incubated with mouse anti-mCherry primary antibody (1:5000, Clontech) and rabbit anti-GFP Alexa Flour 488 conjugated (1:500, Invitrogen) overnight at 4°C. A Cy3-conjugated secondary antibody (1:200, Jackson ImmunoResearch) was used to amplify mCherry. For p-S6 (Ser235/236), p-mTOR (Ser2448) and Pten staining, the sections were stained with chicken anti-GFP (1:1000, abcam), mouse anti-mCherry, and rabbit anti-p-S6 (1:100, Cell Signaling) or rabbit anti-Pten (1:100, Cell Signaling) or rabbit anti-p-mTOR (1:50, Cell Signaling) and incubated overnight at 4°C. A DyLight674 conjugated secondary antibody (1:200, Jackson ImunoResearch) was used to visualize p-S6, p-mTOR, and Pten. For Pten and p-mTOR staining, heat antigen retrieval method was used where slices were bathed in 10mM sodium citrate with 0.5% TWEEN-20 and heated at 90–100°C for 30 min. Cy3, Alexa Flour 488- and DyLight 674-conjugated secondary antibodies were applied (1:200, Jackson ImmunoResearch) overnight at 4°C. Sections were mounted with Vectashield mounting medium with 4′,6-diamidino-2-phenylindole (DAPI, Vector Laboratories).

### Imaging

Image *z*-stacks of granule neurons in the dentate gyrus were taken using a Zeiss LSM 510 laser-scanning confocal microscope. An investigator blinded to the virus status of the animal imaged and analyzed granule neurons from anatomically equivalent sections of the suprapyramidal blade of the dorsal portion of the dentate gyrus. Soma images were taken using a 20× lens with a 3× zoom or a 40× lens with a 1× zoom, at 1024 × 1024 at a 1.3 μm virtual section thickness with a *z*-step of 2 μm.

### Image analysis

An investigator blind to the experimental condition imaged and analyzed granule neurons from anatomically equivalent sections of the suprapyramidal blade of the dorsal portion of the dentate gyrus. In Image J, soma sizes were determined by manually tracing the circumference of each neuron at its thickest point in the z-stack to determine the cross sectional area. Immunohistochemistry (IHC) images were analyzed in by circling somas and measuring the mean gray intensity value in the blue channel for similar numbers of red control and green shRNA neurons. The blue intensity values (representing Pten, p-S6 or p-mTOR immunostaining) obtained from the green Pten knockdown somas were normalized to the average blue intensity obtained from the red control somas. The normalized intensity was calculated by dividing the blue fluorescence intensity of each neuron by the average intensity of the red control neurons for each image stack. Therefore, fluorescence intensity was normalized to control neurons on a per image basis, allowing us to control for variability in staining intensity across different tissues.

### Statistical research design

All values are reported as the mean of all neurons in each group±standard error of the mean. All statistical comparisons (*p*-values) were calculated using the following statistical model unless otherwise indicated in the results. Mice were randomly assigned to rapamycin, fatty acid or vehicle groups and cells within each mouse were randomly infected with an shRNA against Pten or an mCherry control virus. The number of cells extracted from each mouse varied as opposed to being pre-determined, depending on the level of infection of each virus. Soma measurements of each cell were made following delivery of the treatments. However, some of these were only available on a subset of mice. The primary hypothesis is that the effect of Pten is enhanced under fatty acid delivery relative to vehicle delivery. The secondary hypothesis is that Pten knockdown has a significant impact compared to the control treatment under fatty acid delivery (irrespective of the effect of Pten under the control). The secondary hypothesis is weaker in the sense that it does not isolate the extent that the environment impacts the effect of Pten on neuron size.

To isolate the effects and test the hypotheses of interest in the most robust way, we use statistical models that account for the facts that rapamycin or fatty acid treatment is a between mouse factor, while the Pten knockdown is a within-mouse factor, rapamycin or fatty acids and Pten knockdown are suspected of having an interaction effect, and cells are clustered within mice. Such models ensure that statements of statistical precision (confidence intervals and *p*-values) are appropriately calibrated for the fact that observations from the same mouse are statistically dependent and so should not be analyzed as if each cell belonged to a different mouse. As a result, the precision of estimates related to rapamycin or fatty acid treatments are reduced (standard errors are larger) by the extent to which observations on the same mouse are more similar than observations on different mice. In contrast, estimates of the effect of Pten knockdown are estimated with more precision (smaller standard errors) than is a single cell was available from many mice as treatment varies within the same mouse and so the effect of mouse (the random variation in the outcome variable between mice) can be extracted from the comparison of treatments.

When analyzing outcomes measured on more than 5 mice we used mixed-effects linear regression models with a random effect for each mouse; predictors for fatty acids, rapamycin, Pten knockdown, and the interaction of these factors; and robust variance estimation (so that the legitimacy of the inferences are not fully reliant on the correct specification of the model). The requirement of more than 5 mice is imposed because with fewer mice the variability of the outcomes between the mice is so imprecisely estimated there would be concerns over the robustness of the results to the assumptions of the random effect distribution underlying the statistical model. For analyses with fewer than 5 mice the model is structurally identical but we treat the mouse effect as a standard regression coefficient as opposed to as a random draw from the target population of mice. The result is that we obtain slightly more conservative but robust estimates. The mixed effect models (>5 mice) were estimated using the xtmixed procedure while standard regression analyses (≤5 mice) were estimated using the reg procedure in Stata version 13.0. In each case we used the robust option to compute standard errors. As well as the estimated effects and tests of statistical significance of the primary and secondary hypotheses, we also report the estimated mean values averaged across the mice in this study of the outcome under the fitted model for the genetic and pharmacological treatment settings.

## Results

### Lentiviral Pten knockdown results in neuronal hypertrophy

**Figure 1:**
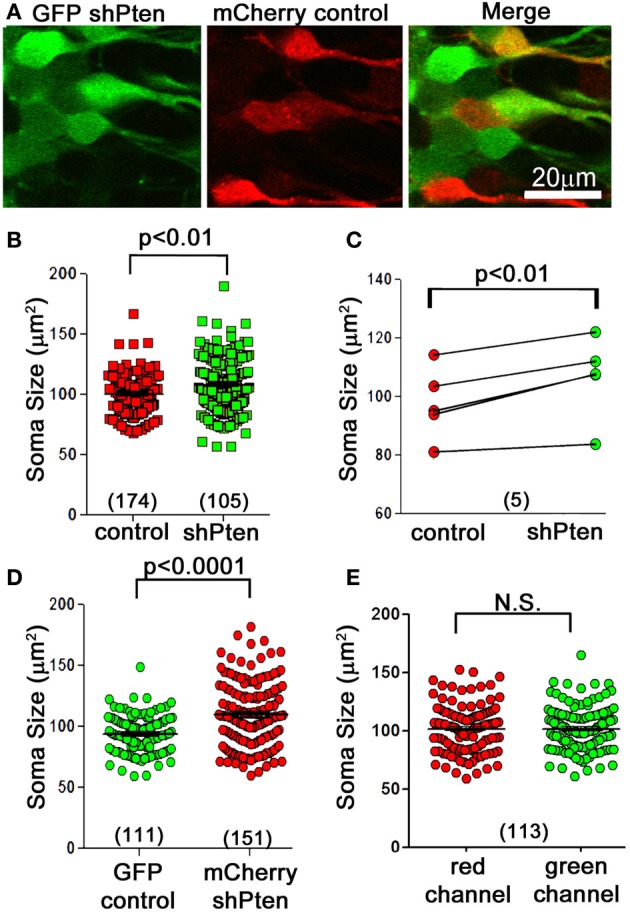
**Neuronal hypertrophy in lentiviral Pten knockdown neurons**. A lentivirus to knock down Pten (GFP shPten; green) and a control lentivirus (mCherry control; red) were co-injected into the dentate gyrus of young adult mice and the morphological results assessed at 21 days post-injection. **(A)** We observed an increase in soma size in Pten knockdown neurons (green) compared to controls (red). **(B)** Plot of soma cross-sectional area of individual control and Pten knockdown neurons. **(C)** Cross-sectional area of control and knockdown neurons plotted as an average per animal. Quantitative analysis of soma cross-sectional area from confocal image stacks revealed that Pten knockdown results in an increase in soma size (108.1 ± 1.66 μm^2^ compared to 100 ± 1.75 μm^2^ in the control; *p* < 0.01, *n* = 174 and 105 neurons from 5 animals). **(D)** Pten knockdown resulted in an increase in soma size regardless of flourophores. Mice were co-injected with mCherry shPten and GFP control viruses, and there was a significant increase in the soma size of mCherry shPten infected neurons compared to GFP infected controls (GFP control = 95.19 ± 1.47 μm^2^, mCherry shPten = 110.9 ± 2.19 μm^2^, *p* < 0.0001, two-tailed *t*-test, *n* = 111 neurons and 151 neurons from 2 animals). **(E)** There was no difference in soma size when the same co-labeled neurons were analyzed in the red or green channel (soma size in red channel = 101.8 ± 1.94 μm^2^, average soma size in green channel = 101.8 ± 1.85 μm^2^, *p* > 0.05, two-tailed *t*-test, *n* = 113 neurons from 3 animals).

C57BL/6 mice at 6–8 weeks of age were co-injected with an FUGW-based lentivirus expressing both GFP and an shRNA targeting the *Pten* coding region (GFP shPten) and a control virus expressing only mCherry (mCherry control; Figure 1A) (Lois et al., 2002; Luikart et al., 2011b). This injection scheme allows us to compare the morphological impact of Pten knockdown to control neurons in the same tissue. At 21 days post-injection the soma size of Pten knockdown neurons was 108.1 ± 1.66 μm^2^ compared to 100 ± 1.75 μm^2^ in the control (Figures 1A–C; *p* < 0.01, *n* = 174 and 105 neurons from 5 animals, see Methods for statistical model). We illustrate this data per neuron (Figure 1B) and as paired data per animal (Figure 1C). For such data statistical significance is determined by taking into account both the number of neurons and the number of animals (see Methods). These data demonstrate that at 21 days post-injection, Pten knockdown results in modest neuronal hypertrophy in adult animals. Previous work indicated that neonatal animals are more sensitive to Pten knockdown with neuronal hypertrophy detectable by 14 days post-injection. However, in adults, no morphological changes were detected at 14 days post-injection and overt hypertrophy was detected by 4 months-post injection (Luikart et al., 2011b). Thus by focusing the present study at 21 days post-injection we are able to focus on the earliest detectable manifestation of the Pten knockdown phenotype.

Since intensity of the different fluorophores as well as confocal laser and detector settings could potentially affect the perceived circumference of cells, we performed two separate experiments to control for these variables. First, we switched the fluorophore of the knockdown and control viruses and measured the somas of neurons infected with an mCherry shPten virus or a GFP control virus. This experiment resulted in a significant increase in soma size for the mCherry knockdown neurons compared to the GFP control neurons (Figure 1D; GFP control = 95.19 ± 1.47 μm^2^, mCherry shPten = 110.9 ± 2.19 μm^2^, *p* < 0.0001, two-tailed *t*-test, *n* = 111 neurons and 151 neurons from 2 animals). We also addressed this issue by measuring the cross sectional area of cells infected with both GFP shPten and mCherry control in the red and green channels, and comparing the measurements from each channel on a per cell basis. A two-tailed *t*-test revealed no statistical difference in the measurements obtained from each channel (Figure 1E; average soma size in red channel = 101.8 ± 1.94 μm^2^, average soma size in green channel = 101.8 ± 1.85 μm^2^, *p* > 0.05, two-tailed *t*-test, *n* = 113 neurons from 3 animals). These control experiments demonstrate that our data is not influenced by differences in fluorophores.

**Figure 2:**
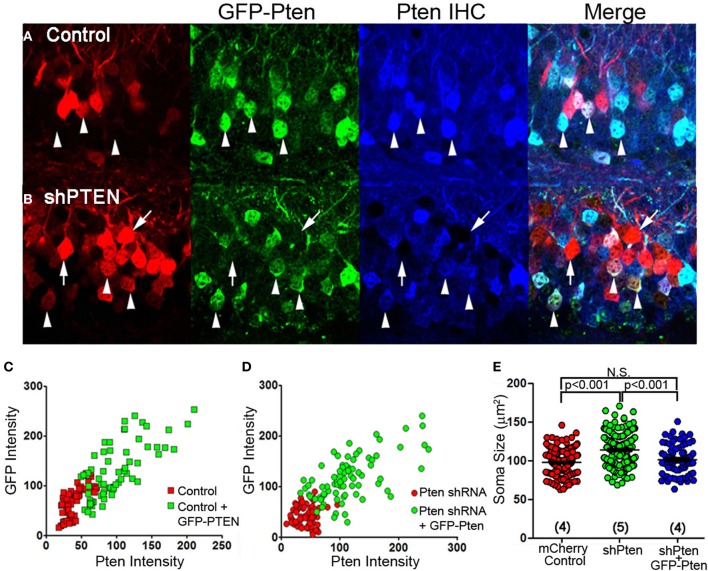
**Over-expression of wild-type Pten in Pten deficient neurons rescues hypertrophy caused by loss of function**. An shRNA-insensitive GFP-Pten (GFP-PTEN) was generated by altering the wobble base pairs of the shRNA target sequence without changing the sequence of the encoded protein. **(A)** Neurons co-injected with an mCherry control virus and GFP-Pten were immuno-stained for Pten. **(B)** We then co-injected an mCherry labeled Pten shRNA and GFP-Pten lentivirus and immuno-stained these neurons for Pten. Neurons that were co-labeled (arrow heads) had an increase in Pten staining intensity compared to those expressing the shRNA only (arrows) **(C)** The GFP-Pten infected neurons (arrow heads) had a higher intensity of Pten staining than the wildtype neurons, and there was a positive correlation between Pten staining intensity and GFP intensity (*R*^2^ = 0.02, linear regression analysis). **(D)** There was a positive correlation between GFP signal and Pten immune-staining intensity in neurons expressing the Pten shRNA (*R*^2^ = 0.051; linear regression analysis). The positive Pten staining in these co-labeled neurons demonstrates that we can knockdown endogenous Pten and replace it with shRNA-insensitive exogenous Pten. **(E)** To assess the functionality of GFP-Pten *in vivo* we analyzed the soma size of these infected neurons. While knocking down Pten increased the soma size of these neurons, cells infected with the Pten shRNA and GFP-Pten had soma sizes that were not significantly different than control cells (mCherry control = 97.8 ± 0.95 μm^2^, vs. shPten = 113.8 ± 1.30 μm^2^, *p* < 0.001, ANOVA, *n* = 234 and 221 neurons from 4 and 5 animals. shPten = 113.8 ± 1.30 μm^2^ vs. shPten + GFP-Pten = 101.3 ± 2.10 μm^2^, *p* < 0.001, ANOVA, *n* = 221 and 82 neurons from 5 and 4 animals. mCherry control vs. shPten + GFP-Pten, *p* > 0.05, ANOVA, *n* = 234 and 82 neurons from 4 animals per group).

The efficacy and specificity of the shRNA used to knockdown Pten in these experiments has previously been characterized (Luikart et al., 2011a). Here, we express an shRNA-insensitive Pten overexpressing virus (GFP-Pten) in knockdown neurons to restore function and provide definitive evidence that our shRNAs do not have off-target effects responsible for the neuronal hypertrophy. We first expressed GFP-Pten in the wild type background by co-injecting mCherry control with GFP-Pten virus and performed anti-Pten immunohistochemistry (Figure 2A). Pten-staining intensity positively correlated with GFP-intensity, demonstrating that greater GFP fluorescence correlated with greater Pten over-expression (Figure 2C). Cells co-infected with mCherry shPten and GFP-Pten also demonstrated positive Pten staining in co-labeled cells compared to cells just infected with shPten (Figure 2B). This positive correlation between GFP intensity and Pten intensity in the Pten shRNA-expressing cells, demonstrates that the GFP-Pten is shRNA-insensitive and can be used to reconstitute Pten into Pten knockdown cells (Figure 2D). Not only did overexpression of Pten restore Pten staining intensity, but it also rescued the increase in soma size seen in Pten knockdown neurons (Figure 2E; mCherry control = 97.8 ± 0.95 μm^2^, vs. shPten = 113.8 ± 1.30 μm^2^, *p* < 0.001, ANOVA, *n* = 234 and 221 neurons from 4 and 5 animals; shPten = 113.8 ± 1.30 μm^2^ vs. shPten + GFP-Pten = 101.3 ± 2.10 μm^2^, *p* < 0.001, ANOVA, *n* = 221 and 82 neurons from 5 and 4 animals; mCherry control vs. shPten + GFP-Pten, *p* > 0.05, ANOVA, *n* = 234 and 82 neurons from 4 animals each). These results indicate that the neuronal hypertrophy associated with Pten shRNA expression is due solely to loss of Pten expression.

### p-S6 and p-mTOR correlate to increased soma size in Pten knockdown neurons

**Figure 3:**
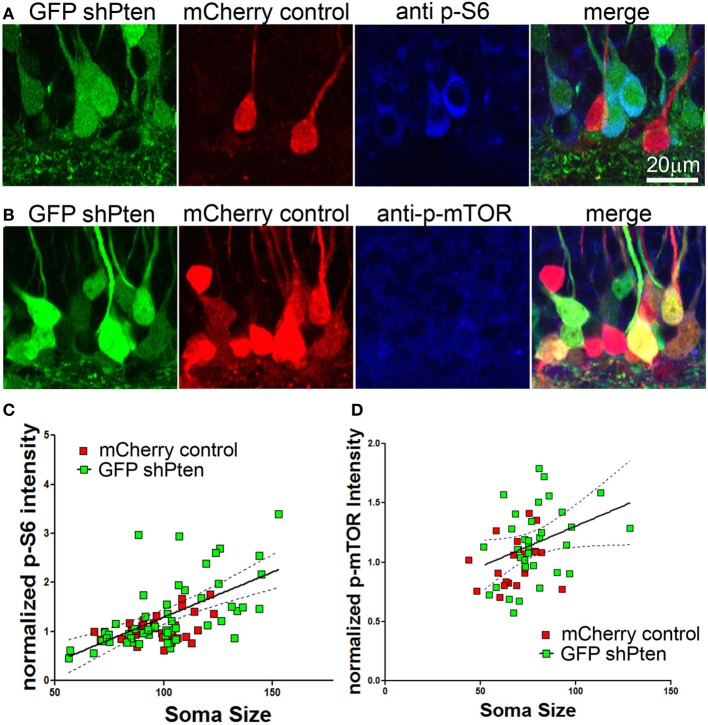
**p-S6/p-mTOR correlated to increased soma size in Pten knockdown neurons**. Adult animals were co-injected with the Pten knockdown lentivirus (GFP shPten; green) and the mCherry expressing control virus (mCherry control; red) and immunohistochemistry was used to evaluate p-S6 or p-mTOR expression (blue) at 21 days post-injection. **(A,B)** Increased p-S6 or p-mTOR staining (blue) was observed in the GFP shPten cells (green) when compared to neighboring mCherry control cells (red) in the same histological section. **(C)** The fluorescence intensity of the p-S6 staining was measured and normalized to the average value obtained for control cells in each histological section. A linear regression analysis was then used to demonstrate that there is a positive correlation between p-S6 staining intensity and soma size. A best fit line for the Pten knockdown neurons (solid) is shown with the 95% confidence interval (dashed) (slope = 0.018 ± 0.003; *R*^2^ = 0.3734, *p* < 0.001) **(D)** Similar to p-S6, an increase in the p-mTOR staining intensity (blue) was observed in Pten knockdown neurons (green). A scatter plot of soma size x normalized p-mTOR intensity indicates a positive correlation between soma size and p-mTOR intensity in the Pten knockdown neurons (*R*^2^ = 0.121; *p* < 0.01; solid line, best fit; dashed line, 95% confidence interval; linear regression analysis).

We next examined whether this neuronal hypertrophy correlated with increased downstream signaling in response to Pten knockdown. We assessed downstream signaling by normalizing the immunofluorescence intensity of p-S6 or p-mTOR in Pten knockdown cells to neighboring control cells (Figure 3). Although this method allows relative comparison of fluorescence intensity on a per-cell basis, it does not allow quantitation of absolute values because a standardized linear range of detection cannot be established. We found that there was an increase in p-S6 staining intensity in Pten knockdown neurons when compared to neighboring control neurons (Figure 3A, mCherry control = 1.00 ± 0.05, GFP shPten = 1.33 ± 0.10, *p* < 0.05, *t*-test, *n* = 34 and 54 neurons from 2 animals). We next used a linear regression analysis to examine whether there was a correlation between soma size and p-S6 intensity on a per cell basis (Figure 3C). In control neurons, we found a positive correlation between p-S6 staining intensity and soma size (slope = 0.0077 ± 0.003, *R*^2^ = 0.1678, *p* < 0.05). We also found a positive slope for the linear regression for soma size x p-S6 intensity of 0.018 ± 0.003 in the Pten knockdown neurons (Figure 3C; *R*^2^ = 0.3734, *p* < 0.001). A comparison of the slopes between the control and the Pten knockdown neurons did not reveal a difference (*p* = 0.097). Thus the relationship of soma size to p-S6 intensity was similar between control neurons and Pten knockdown neurons. However, the magnitude of the signaling response and associated increase in soma size was increased in Pten knockdown neurons when compared to controls.

Similarly, there was an increase in p-mTOR in Pten knockdown neurons (Figure 3B; mCherry control = 1.00 ± 0.04, GFP shPten = 1.19 ± 0.06, *p* < 0.05, *t*-test, *n* = 21 and 34 neurons from 2 animals). Again the slope of the linear fit of the data indicated a positive correlation between p-mTOR staining intensity and soma size (mCherry control = 0.0045 ± 0.0039, *R*^2^ = 0.068; GFP shPten = 0.0068 ± 0.0033, *R*^2^ = 0.121). However, this positive correlation was only significant in the Pten knockdown neurons (Figure 3D; mCherry control *p* = 0.254, GFP shPten *p* = 0.0472). Thus while Pten knockdown resulted in an increase in p-mTOR staining, there was a stronger correlation of soma size to p-S6 staining than for p-mTOR. It is important to note that an antigen retrieval procedure must be used to stain for p-mTOR and we found that this procedure results in tissue shrinkage and an overall decrease in soma size measurements. Thus immunohistochemical staining for p-S6 is a more robust assay - displaying better preservation of morphology, larger dynamic range, and improved signal to background ratio than that achieved for p-mTOR.

### Fatty acids increase the effects of Pten knockdown on adult neurons

**Figure 4:**
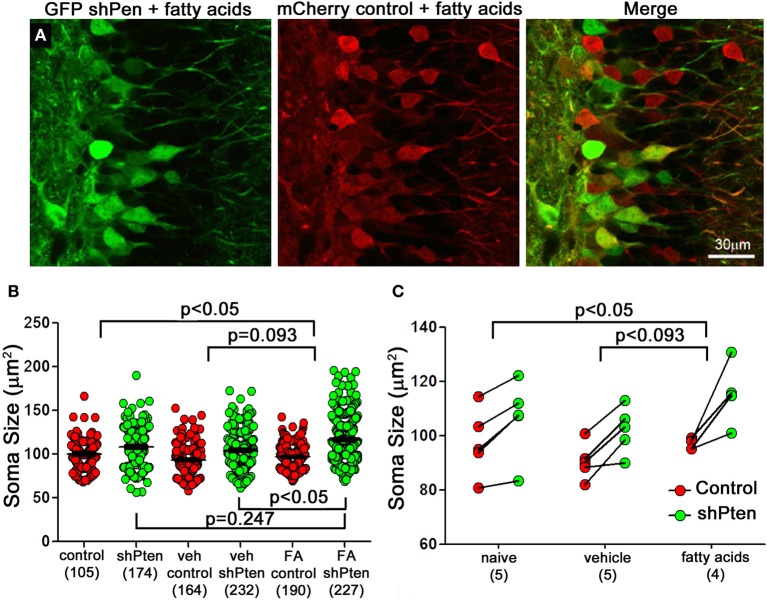
**Fatty acids increase neuronal hypertrophy in Pten knockdown neurons. (A)** Adult mice were injected with the mCherry control (red) and the GFP shPten (green) lentiviruses. At seven days post-injection they were implanted with mini- pumps containing a mixture of palmitic, palmitoleic, and myristic acid. Fatty acid treated knockdown neurons were significantly larger than their neighboring controls (FA shPten = 116.6 ± 1.50 μm^2^, FA control = 96.9 ± 1.04 μm^2^, *p* < 0.001, *n* = 227 and 190 neurons from 4 animals). **(B)** Soma size is displayed for individual control and knockdown neurons in the naïve, vehicle, and fatty acid treated groups. Pten knockdown resulted in neuronal hypertrophy regardless of treatment (naïve control = 100 ± 1.75 μm^2^, naïve shPten = 108.1 ± 1.66 μm^2^, *p* < 0.001, *n* = 105 and 174 neurons from 5 animals; Veh control = 93.1 ± 1.31 μm^2^, Veh shPten = 104.1 ± 1.38 μm^2^, *p* < 0.001, *n* = 164 and 232 neurons from 5 animals; FA control = 96.9 ± 1.04 μm^2^, FA shPten = 116.6 ± 1.5 μm^2^, *p* < 0.001, *n* = 190 and 227 neurons from 4 animals) and fatty acid treatment exacerbated this effect (veh shPten vs. FA shPten, *p* < 0.05). **(C)** Displaying the data grouped by animal demonstrates an increase in soma size of the fatty acid treated neurons compared to wild type (naïve control = 100 ± 1.75 μm^2^, naïve shPten = 108.1 ± 1.66 μm^2^ vs. FA control = 96.9 ± 1.04 μm^2^, FA shPten = 116.6 ± 1.5 μm^2^, *p* < 0.05, *n* = 105, 174, 190, and 227 neurons from 5 and 5 animals). However, comparison of the change in soma size produced by Pten in the fatty acid treatment group and the vehicle treated was not quite significant (Veh control = 93.1 ± 1.31 μm^2^, Veh shPten = 104.1 ± 1.38 μm^2^ vs. FA control = 96.9 ± 1.04 μm^2^, FA shPten = 116.6 ± 1.5 μm^2^, *p* = 0.093, *n* = 164, 232, 290, and 227 neurons from 5 and 5 animals). Overall, these data demonstrate a trend toward increased hypertrophy of knockdown cells in the fatty acid treated cells compared to the knockdown cells in the vehicle and naïve controls.

Increasing levels of circulating fatty acids can activate the p-Akt/p-mTOR pathway in hepatocytes and cardiomyocytes (Cao et al., 2008; Riquelme et al., 2011). To examine the effect of fatty acids on Pten knockdown neurons we first injected the mCherry control and GFP shPten viruses into adult mice and implanted subcutaneous mini osmotic pumps containing a mixture of palmitoleic, myristic, and palmitic acids (1:6:16) one week later(Riquelme et al., 2011). Ten days post-implantation, fatty acid treatment resulted in an increase in the effect of Pten knockdown on soma size when compared to naïve animals (Figures 4A–C; naïve control = 100 ± 1.75 μm^2^, naïve shPten = 108.1 ± 1.66 μm^2^ vs. FA control = 96.9 ± 1.04 μm^2^, FA shPten = 116.6 ± 1.5 μm^2^, *p* < 0.05, *n* = 105, 174, 190, and 227 neurons from 5 animals per group). This difference can be appreciated by observing the net change in soma size caused by the Pten knockdown as depicted with all neurons examined (Figure 4B) or with the data grouped by animal (Figure 4C). The change in soma size produced by Pten knockdown in the fatty acid treatment group approaches significance when compared to the vehicle treated group (Veh control = 93.1 ± 1.31 μm^2^, Veh shPten = 104.1 ± 1.38 μm^2^ vs. FA control = 96.9 ± 1.04 μm^2^, FA shPten = 116.6 ± 1.5 μm^2^, *p* = 0.093, *n* = 164, 232, 190, and 227 neurons from 5 animals per group). Pairwise comparisons reveal that fatty acid treated Pten knockdown neurons are larger than knockdown neurons in the vehicle (FA shpten = 116.6 ± 1.5 μm^2^, vs. Veh shPten = 104.1 ± 1.38 μm^2^, *p* < 0.05, *n* = 227 and 232 neurons from 5 animals per group). While the amplitude of this change was similar when comparing the fatty acid treated animals to the naïve animals, the increased variability in the naïve shPten animals masked this effect (naïve shPten = 108.1 ± 1.5 μm^2^, vs. FA shPten = 116.6 ± 1.5 μm^2^, *p* > 0.05, *n* = 174 and 227 neurons from 5 animals per group). Thus fatty acid treatment produced a modest increase in soma size of Pten knockdown neurons when compared to the naïve and vehicle treated animals.

### Fatty acids increase p-S6 signaling in Pten knockdown neurons

**Figure 5:**
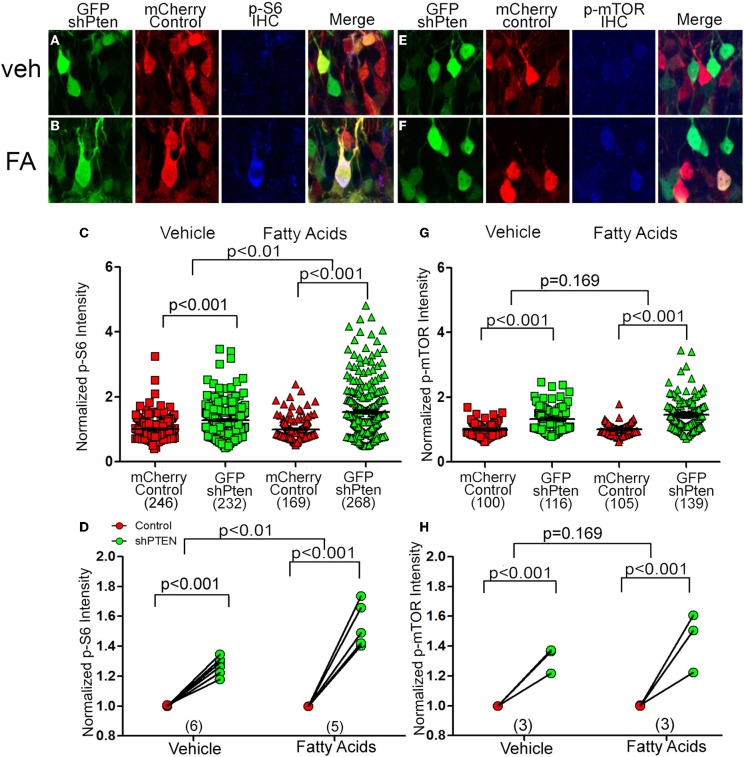
**Circulating fatty acids increase p-S6 and p-mTOR signaling in Pten knockdown neurons**. Adult animals were co-injected with the Pten knockdown lentivirus (GFP shPten; green) and the mCherry expressing control virus (mCherry control; red). Mini-osmotic pumps were implanted seven days post-injection and then seven days after implantation immunohistochemistry was used to evaluate p-S6 or p-mTOR expression (blue). **(A)** A slight increase in p-S6 expression (blue) was detected in Pten knockdown neurons (green) when compared to control neurons (red) in vehicle treated animals. **(B)** In animals treated with fatty acids, a marked increase in p-S6 staining (blue) was observed in the GFP shPten cells (green) when compared to the mCherry control cells (red only). **(C)** Plot of individual p-S6 intensities for each knockdown and control neuron normalized to the p-S6 intensity in controls. **(D)** Pten knockdown resulted in an increase in p-S6 signaling in knockdown neurons compared to controls in the vehicle control as well as the fatty acid treatment groups (mCherry control Veh = 1.0 ± 0.02, GFP shPten Veh = 1.28 ± 0.03, *p* < 0.001, *n* = 246 and 232 neurons from 6 animals. mCherry control FA = 1.0 ± 0.02, GFP shPten FA = 1.54 ± 0.05, *p* < 0.001, *n* = 169 and 268 neurons from 5 animals). There was a significant increase in the p-S6 intensity of fatty acid treated neurons compared to vehicle neurons (*p* < 0.01) demonstrating that fatty acids increased p-S6 signaling beyond what was seen with Pten knockdown. **(E)** Similar to p-S6, an increase in the p-mTOR staining intensity (blue) was observed in Pten knockdown neurons (green) vs. mCherry control neurons (red) in the vehicle treated group. **(F)** p-mTOR activation was more apparent in the Pten shRNA neurons (green) when compared to control neurons (red) after fatty acid treatment. **(G)** Plot of individual p-mTOR intensities for each knockdown and control neuron normalized to the p-mTOR intensity in controls. **(H)** Pten knockdown resulted in an increase in p-mTOR signaling in knockdown neurons compared to controls in the vehicle and fatty acid treatments (mCherry control Veh = 1.0 ± 0.02, GFP shPten Veh = 1.32 ± 0.03, *p* < 0.001, *n* = 100 and 116 from 3 animals; mCherry control FA = 1.0 ± 0.02, GFP shPten FA = 1.46 ± 0.04, *p* < 0.001, *n* = 105 and 139 from 3 animals). However, there was no significant difference between the vehicle and fatty acid treated neurons (*p* = 0.169).

To determine whether fatty acids altered signaling downstream of Pten we injected the control and shPten viruses into 7 week old animals and implanted mini-osmotic pumps containing palmitoleic, myristc, and palmitic acids 7 days post injection. There was a significant increase in p-S6 staining in Pten knockdown neurons in the vehicle controls as well as the fatty acid treatment groups (Figures 5A–D; mCherry control Veh = 1.0 ± 0.02, GFP shPten Veh = 1.28 ± 0.03, *p* < 0.001, *n* = 246 and 232 from 6 animals. mCherry control FA = 1.0 ± 0.02, GFP shPten FA = 1.54 ± 0.05, *p* < 0.001, *n* = 169 and 268 from 5 animals). Also, there was a larger increase in p-S6 staining intensity in the fatty acid treated mice than in the vehicle mice (*p* < 0.01). Thus, fatty acid treatment significantly increased p-S6 staining in Pten knockdown neurons compared to vehicle.

Like p-S6, there was a significant increase in p-mTOR between control neurons and knockdown neurons in the vehicle controls as well as the fatty acid treatment groups (Figures 5E–G; mCherry control Veh = 1.0 ± 0.02, GFP shPten Veh = 1.32 ± 0.03, *p* < 0.001, *n* = 100 and 116 from 3 animals per group. mCherry control FA = 1.0 ± 0.02, GFP shPten FA = 1.46 ± 0.04, *p* < 0.001, *n* = 105 and 139 from 3 animals per group). There was a trend toward this increase being greater in the fatty acid treated cells (Figures 5G,H, *p* = 0.169). As a whole these data indicate that increases in circulating fatty acids result in increased neuronal hypertrophy and p-S6 signaling in Pten knockdown neurons.

### Rapamycin inhibits neuronal hypertrophy and increased p-S6 signaling in Pten knockdown neurons

**Figure 6:**
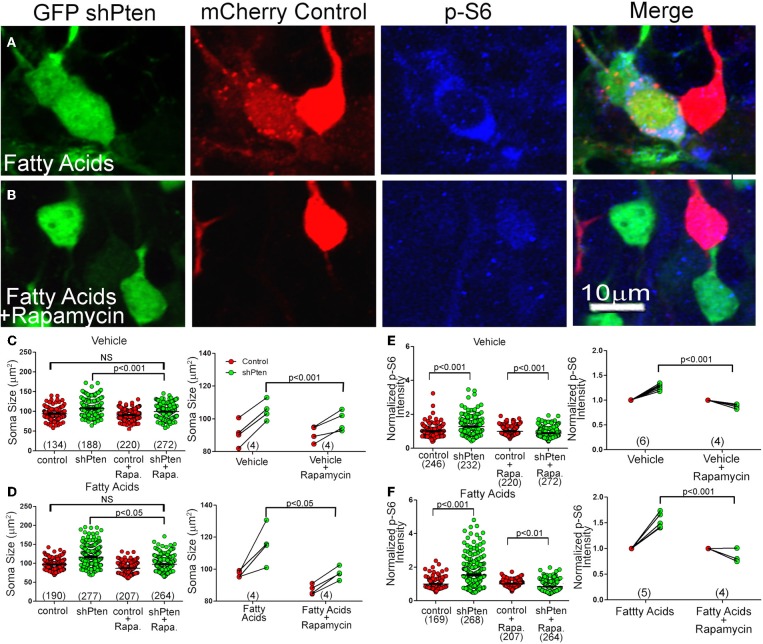
**Rapamycin Inhibits Neuronal Hypertrophy and Increased p-S6 signaling Caused by Pten Knockdown (A)** Pten knockdown neurons treated with fatty acids (green) have an increase in soma size and p-S6 intensity (blue) compared to neighboring control neurons (red). **(B)** Treating these fatty acid exposed, Pten knockdown neurons with rapamycin (green) inhibits this increase in soma size and increase in p-S6 staining (blue) compared to control neurons (red). **(C)** In vehicle treated neurons, there is still an increase in soma size when comparing the knockdown neurons to control neurons (Vehicle control = 94.13 ± 1.36, Vehicle shPten = 107.4 ± 1.52, *p* < 0.001, *n* = 134 and 188 neurons from 4 animals). There is also still an increase in soma size when comparing the knockdown neurons to the control neurons in the rapamycin treated cells (Veh control + rapa = 91.03 ± 0.86, Veh shPten + rapa = 99.36 ± 1.00, p < 0.001, *n* = 220 and 272 cells from 4 animals). However, there is no difference between the Veh shPten + rapa cells and the Veh control cells (*p* > 0.05), showing that rapamycin was able to rescue the soma size of knockdown cells to that of the untreated controls. There is also a significant difference between Veh shPten cells and the Veh shPten + rapa cells (*p* < 0.001, right panel), demonstrating that rapamycin treatment reduced the size of Pten knockdown neurons. **(D)** Like the vehicle, there is also still a significant increase in soma size in fatty acid treated Pten knockdown neurons compared to their controls in the rapamycin treated and non-treated groups (FA control = 96.86 ± 1.04, FA shPten = 116.6 ± 1.50, *p* < 0.001, *n* = 190 and 277 neurons from 4 animals; FA control + rapa = 87.23 ± 0.94, FA shPten + rapa = 97.38 ± 1.23, *p* < 0.001, *n* = 207 and 264 neurons from 4 animals). There was no significant difference between fatty acid treated control neurons and fatty acid treated Pten knockdown neurons treated with rapamycin, demonstrating that even with fatty acid treatment, rapamycin is still able to rescue the soma size of knockdown neurons to that of controls (FA control vs. FA shPten + rapa, *p* > 0.05). Finally, there was a significant difference between fatty acid treated Pten knockdown neurons and fatty acid treated Pten knockdown neurons treated with rapamycin (FA shPten vs. FA shPten + rapa, *p* < 0.05, right panel), demonstrating that rapamycin is able to rescue the soma size of fatty acid treated Pten knockdown neurons. **(E)** In vehicle treated animals, there is an increase in p-S6 signaling in knockdown neurons compared to controls (Veh control = 1.00 ± 0.02, Veh shPten = 1.28 ± 0.03, *p* < 0.001, *n* = 246 and 232 neurons from 6 animals). With rapamycin treatment, there is a reversal of this effect and a robust decrease in p-S6 signaling in the knockdown cells (Veh control + rapa = 1.00 ± 0.02, Veh shPten + rapa = 0.89 ± 0.02, *p* < 0.001, *n* = 220 and 272 neurons from 4 animals). Therefore, there is a significant difference in p-S6 signaling in vehicle rapamycin treated animals and untreated animals (*p* < 0.001). **(F)** Again, there is an increase in p-S6 signaling in fatty acid treated knockdown neurons compared to controls (fatty acid control = 1.0 ± 0.02, fatty acid shPten = 1.54 ± 0.05, *n* = 169 and 268 neurons from 5 animals). However, even with fatty acid treatment, there is still a significant decrease in p-S6 staining in rapamycin treated knockdown neurons compared to controls (FA control + rapa = 1.00 ± 0.01, FA shPten + rapa = 0.85 ± 0.02, *p* < 0.01, *n* = 207 and 264 neurons from 4 animals). Therefore, there is a significant decrease in p-S6 signaling in fatty acid and rapamycin treated neurons compared to fatty acid treatment alone (right, *p* < 0.001).

Since we found increased p-S6 and p-mTOR signaling in Pten knockdown neurons, we hypothesized that rapamycin, a potent mTOR inhibitor, would reverse or inhibit this neuronal hypertrophy. We again co-injected adult mice with mCherry control and GFP shPten viruses and implanted mini-osmotic pumps to deliver fatty acids. After implantation of the pumps, we treated mice with daily intraperitoneal injections of rapamycin for 10 days. Rapamycin treatment decreased the size of Pten knockdown neurons compared to untreated Pten knockdown neurons (Figure 6C; Veh shPten = 107.4 ± 1.52 μm^2^, Veh shPten + rapa = 99.36 ± 1.00 μm^2^, *p* < 0.001, *n* = 188 and 272 neurons from 4 animals per group). Further, rapamycin treated Pten knockdown neurons were essentially the same size as wild type neurons (Figure 6C; Veh control = 94.13 ± 1.36, Veh shPten + rapa = 99.36 ± 1.00, *p* = 0.119, *n* = 134 and 272 neurons from 4 animals per group) demonstrating that inhibiting mTOR prevents this neuronal hypertrophy and restores soma size to that of the controls.

Rapamycin was also capable of decreasing the somal hypertrophy of the Pten knockdown neurons of fatty acid treated mice (Figure 6D; fatty acid shPten = 116.6 ± 1.50 μm^2^, fatty acid shPten + rapa = 97.38 ± 1.23 μm^2^, *p* < 0.05, *n* = 277 and 264 neurons from 4 animals each). The rapamycin treatment of Pten knockdown cells compared to fatty acid treated control cells reveals no difference in soma size (Figure 6D; FA shPten + rapa = 97.38 ± 1.23 μm^2^, FA control = 96.86 ± 1.04, *p* = 0.454, *n* = 190 and 264 neurons from 4 animals each), showing that rapamycin was capable of restoring the most hypertrophic cells to the size of wild-type neurons.

The effect of rapamycin on p-S6 levels was more robust than on soma size. There was actually a decrease in p-S6 signaling in rapamycin treated knockdown neurons compared to rapamycin treated controls (Figure 6E, left; Veh control + rapa = 1.00 ± 0.02, Veh shPten + rapa = 0.089 ±.02, *p* < 0.001, *n* = 220 and 272 neurons from 4 animals per group). There was also a decrease in p-S6 signaling in rapamycin treated knockdown cells compared to non-treated knockdown cells (Figure 6E, right; Veh shPten = 1.28 ± 0.03, Veh shPten + rapa = 0.89 ± 0.02, *p* < 0.001, *n* = 232 neurons from 6 animals and 272 neurons from 4 animals). Therefore, rapamycin eliminated the increase in p-S6 signaling produced by Pten knockdown. In vehicle treated animals, Pten knockdown resulted in an increase of p-S6 staining intensity from 1.00 ± 0.02 to 1.28 ± 0.03 (*p* < 0.001). Rapamycin treated Pten knockdown neurons had a decrease in p-S6 staining when compared to wild type neurons (*p* < 0.001). Even with the addition of fatty acids, rapamycin was still able to decrease the level of p-S6 signaling in knockdown cells compared to neighboring controls (Figures 6A,B,F, right; FA shPten = 1.54 ± 0.05, FA shPten + rapa = 0.85 ± 0.02, *p* < 0.001, *n* = 268 and 264 neurons from 5 and 4 animals respectively). Therefore, rapamycin treatment is able to prevent increases in p-S6 signaling caused by Pten knockdown in both vehicle treated and fatty acid treated animals. This provides evidence that this phenotype is mediated through the mTOR pathway.

### Dietary fatty acids have no effect on Pten knockdown phenotypes

**Figure 7:**
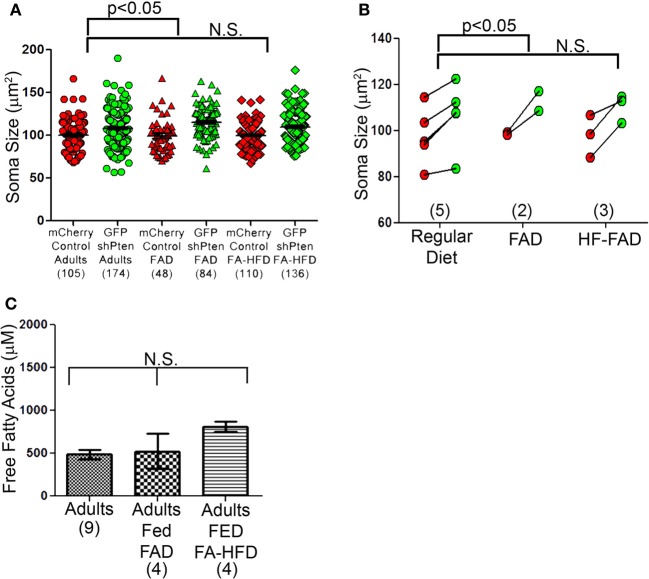
**Dietary fatty acid manipulations do not alter neuronal size. (A)** Three diet groups displayed by neuron show a significant increase in the FAD compared to the control diet, but not the FA-HFD compared to the control diet. **(B)** Soma sizes of Pten knockdown neurons did not differ between adult mice fed a normal diet, a fatty acid adjusted diet (FAD) or a fatty acid adjusted high fat diet (FA-HFD). However, there was still an increase in soma size between the Pten knockdown and control neurons and previously seen (normal chow mCherry control = 100 ± 1.75 μm^2^, normal chow GFP shPten = 108.1 ± 1.66 μm^2^, *p* < 0.001, *n* = 105 and 174 neurons from 5 animals. FAD mCherry control = 99.0 ± 3.00 μm^2^, GFP shPten FAD = 115.0 ± 2.05 μm^2^, *p* < 0.001, *n* = 48 and 84 neurons from 2 animals. mCherry control FA-HFD = 99.8 ± 1.40 μm^2^, GFP shPten FA- HFD = 109.8 ± 1.63 μm^2^, *p* < 0.01, *n* = 110 and 136 neurons from 3 animals). For the FA-HFD, there was no evidence for an increase in soma size further than Pten knockdown alone, but there was a significant increase in the FAD (regular chow vs. FAD, *p* < 0.05) **(C)** Levels of free fatty acids (FFA) were measured in the serum of adults fed normal chow, the FAD, and the FA-HFD. None of the diet manipulations increased the circulating FFA levels of the adults (Adult fed regular chow = 486.8 ± 53.94 μM FFA, Adult fed FAD = 525.6 ± 206.6 μM FFA, Adult fed FA- HFD = 811.6 ± 56.23 μM FFA, *p* > 0.05, one-way ANOVA, *n* = 9, 4, and 4 animals, respectively).

We next examined the effects of dietary fatty acid manipulation on Pten knockdown neurons. We injected 7 week old mice with the mCherry control and the GFP shPten lentiviruses, then placed the animals on either normal rodent chow, a fatty acid adjusted diet (FAD) containing 7.2% fat by weight with palmitoleic:myristic:palmitic acids in the ratio 1:6:16 (modified AIN-93G, Harlan Laboratories), or a fatty acid adjusted high fat diet (FA-HFD) containing 20.3% fat with palmitoleic:myristic:palmitic acids in ratio 1:6:16 (TD.120122, Harlan Laboratories). At 21 days post-injection Pten knockdown increased soma size for all three diets examined (Figure 7A; normal chow mCherry control = 100 ± 1.75 μm^2^, normal chow GFP shPten = 108.1 ± 1.66 μm^2^, *p* < 0.001, *n* = 105 and 174 neurons from 5 animals. FAD mCherry control = 99.0 ± 3.00 μm^2^, GFP shPten FAD = 115.0 ± 2.05 μm^2^, *p* < 0.001, *n* = 48 and 84 neurons from 2 animals. mCherry control FA-HFD = 99.8 ± 1.40 μm^2^, GFP shPten FA- HFD = 109.8 ± 1.63 μm^2^, *p* < 0.01, *n* = 110 and 136 neurons from 3 animals). There was no change in soma size of control neurons in response to diet. Interestingly, the FAD significantly increased the soma size of knockdown neurons over that of the regular diet knockdown neurons (*p* < 0.05, Figure 7B), but there was no increase in hypertrophy of the HF-FAD knockdown neurons. We examined the levels of free fatty acids in the blood of these animals and found that neither the FAD nor the FA-HFD significantly increased the level of circulating fatty acids in the adults (Figure 7C; Adult fed regular chow = 486.8 ± 53.94 μM FFA, Adult fed FAD = 525.6 ± 206.6 μM FFA, Adult fed FA-HFD = 811.6 ± 56.23 μM FFA; *p* > 0.05, One-Way ANOVA, *n* = 9, 4, and 4 animals, respectively). Overall wild-type animals were resistant to fluctuations in circulating fatty acid levels caused by dietary changes and these diets did not affect the phenotypic expression of Pten knockdown.

## Discussion

By co-injecting a control virus with an shRNA-based Pten knockdown virus we were able to compare the effects of Pten knockdown between neurons in the same animal. Using this system we can measure the morphological effects of Pten knockdown and compare immunohistochemical staining intensities by normalizing to levels seen in control neurons within the same histological section. This allows us to eliminate variability in staining that occurs across sections. With such a tightly-controlled system we were able to detect neuronal hypertrophy by 21 days post-injection. Further, the neuronal hypertrophy correlated with increased p-S6 and p-mTOR in individual neurons and was inhibited with rapamycin treatment.

Using this system we chose to determine whether circulating fatty acids could alter the expression of the Pten knockdown phenotype. Circulating fatty acids are able to pass through the blood-brain barrier, so we hypothesized that they could have an effect on neurons (Mitchell and Hatch, 2011). Manipulating the amount of circulating palmitic, palmitoleic, and myristic acids induces cardiac hypertrophy through mTOR signaling (Riquelme et al., 2011). We find that the introduction of this mixture of fatty acids results in a larger increase in soma size in Pten knockdown neurons than seen in naïve adults or vehicle controls. Further the combination of fatty acid infusion and Pten knockdown resulted in greater increases in p-S6 than seen in vehicle controls.

The physiological relevance of experiments involving the direct infusion of fatty acids into animals is unclear. We therefore attempted to manipulate fatty acid levels through diet. The FAD in which the composition of fatty acids was altered but the overall fat content was similar to the normal rodent chow resulted in an increase in soma size in Pten knockdown neurons similar to direct fatty acid infusion. However, the fatty acid adjusted high fat diet with both increased fat content and altered fatty acid composition did not have an effect on soma size when compared to normal chow. Interestingly, neither of these dietary manipulations significantly elevated the levels of circulating fatty acids presumably because metabolic processes regulate levels of circulating fatty acids despite fluctuations in dietary intake. Such metabolic regulation may be affected in individuals with obesity or diabetes such that dietary fat intake results in significant fluctuations of circulating fatty acid levels (Guo et al., 1999; Labbe et al., 2011). Thus chronic dietary manipulation or dietary manipulation in obese/diabetic models may reveal an interaction with Pten knockout.

Although metabolic mechanisms regulating endogenous free fatty acid levels are complicated, experimental elevation of these levels in adult animals enhanced the effects of Pten knockdown. There are a number of potential mechanisms by which fatty acid elevation could mediate this effect. Insulin is able to promote phosphorylation and activation of AKT/mTOR (Scott et al., 1998). Although fatty acids can suppress or potentiate insulin secretion depending on the specific fatty acid species and experimental context (Yaney and Corkey, 2003), it has been suggested that increasing levels of fatty acids could promote growth by regulating insulin sensitivity (Riquelme et al., 2011). Increasing fatty acids could also affect the production of arachidonic acid or prostaglandin E2, both of which can increase mTOR signaling (Kuehn et al., 2011; Wen et al., 2012). Finally, it has been speculated that palmitic acid could activate the PI3K pathway directly via an unknown receptor (Pu et al., 2011).

Although our data do not address the exact mechanisms by which fatty acids potentiate growth signaling, we do demonstrate that we are able to inhibit the hypertrophy and p-S6 signaling with rapamycin, an mTOR inhibitor. Interestingly rapamycin reduced p-S6 levels in Pten knockdown neurons to the levels less than that of control neurons. This could indicate other processes at work to compensate against the increased downstream signaling in the Pten knockdown neurons. We also demonstrate a molecular link between increased fatty acid levels and the neuronal hypertrophy caused by Pten knockdown through the elevation of p-S6/p-mTOR. However, the overall magnitude of this effect is quite small and the dietary manipulation data is inconsistent. It remains to be determined whether long-term fatty acid manipulations could produce a more robust effect and whether this effect is relevant to the pathophysiology of Autism Spectrum Disorder.

### Conflict of interest statement

The authors declare that the research was conducted in the absence of any commercial or financial relationships that could be construed as a potential conflict of interest.
